# THE EFFECT OF FAMILY RELATIONS AND THE SATISFACTION OF SOCIAL ACTIVITIES BY GENDER

**DOI:** 10.1093/geroni/igae098.2483

**Published:** 2024-12-31

**Authors:** Meeryoung Kim

**Affiliations:** Daegu University, Seoul, Republic of Korea

## Abstract

The retirement age in Korea is 60, and most of the time after retirement is spent at home. Sometimes staying at home can be a source of conflict. However, doing social activities and being satisfied with such activities can become energy in old age. From an ecological point of view, this study investigates family relationships and satisfaction with various social relationships and activities as factors influencing the life satisfaction of older adults. The effects of these relationships may vary by gender. This study used the seventh additional and eighth wave of the Korean Retirement and Income Study. The subjects of this study were adults aged 60 and older. The sample size of men is 232 and the sample of women is 165. Multiple regressions were used for data analysis. Demographic variables were controlled. As for independent variables, this study used: the degree of spousal conflict, conflicts with children who reside at home and outside, satisfaction with leisure, and organizational and relational activities. Life satisfaction was used as a dependent variable. Research findings showed that relationship satisfaction had a significant effect on life satisfaction for both men and women. However, relationship satisfaction was relatively higher for men than for women. Unlike men, women’s life satisfaction was impacted negatively by conflict between a spouse and children living together. These results imply that the same conflicts between spouses and children have different effects on life satisfaction by gender difference. Therefore, we must consider gender difference when deciding on old age policy.

